# Adult Erythroblastic Sarcoma With *PCM1::JAK2* Fusion and a Novel *NOP10::NUTM1* Fusion Complicated by Secondary HLH

**DOI:** 10.1111/jcmm.71286

**Published:** 2026-07-15

**Authors:** Tong Ge, Dong Kuang, Xia Mao, Songya Liu, Jin Wang, Qilin Ao, Min Xiao

**Affiliations:** ^1^ Institute of Pathology, Tongji Hospital, Tongji Medical College Huazhong University of Science and Technology Wuhan Hubei China; ^2^ Department of Pathology, School of Basic Medical Science, Tongji Medical College Huazhong University of Science and Technology Wuhan Hubei China; ^3^ Department of Hematology, Tongji Hospital, Tongji Medical College Huazhong University of Science and Technology Wuhan Hubei China; ^4^ Immunotherapy Research Center for Hematologic Diseases of Hubei Province Wuhan Hubei China; ^5^ Key Laboratory of Vascular Aging, Ministry of Education, Tongji Hospital, Tongji Medical College Huazhong University of Science and Technology Wuhan Hubei China

**Keywords:** erythroblastic sarcoma, hemophagocytic lymphohistiocytosis, NUTM1 rearrangement, PCM1::JAK2 fusion

## Abstract

Erythroblastic sarcoma is a rare and aggressive hematologic malignancy presenting as a mass‐forming extramedullary proliferation of immature erythroid cells. Myeloid/lymphoid neoplasms with JAK2 rearrangement, most often PCM1::JAK2, may show eosinophilia, myelofibrosis, and expansion of immature erythroid precursors. NUTM1 rearrangements, initially recognized as defining alterations of NUT carcinoma, have subsequently been identified in selected hematologic malignancies but remain exceptionally rare in myeloid neoplasms. Here, we report an adult erythroblastic sarcoma harbouring concurrent PCM1::JAK2 and a novel NOP10::NUTM1 fusion, accompanied by partial chromosome 8p deletion and complicated by secondary hemophagocytic lymphohistiocytosis. To our knowledge, this is the first reported adult erythroblastic sarcoma with concurrent PCM1::JAK2 and NOP10::NUTM1 fusions. This case extends the molecular spectrum of erythroblastic sarcoma and underscores the value of integrated morphologic, immunophenotypic, cytogenetic, and genomic assessment in diagnostically challenging erythroid neoplasms.

## 1 Introduction

Erythroblastic sarcoma (ES) is an exceedingly rare, mass‐forming proliferation of immature erythroid cells that may occur with or without overt acute erythroid leukaemia (AEL) [1]. Because the number of reported cases remains limited, its diagnostic criteria, immunophenotypic spectrum, and molecular landscape remain incompletely defined [2]. Adult ES is often associated with high‐risk genetic features, including biallelic TP53 alterations and complex karyotypes, and generally carries an adverse prognosis [3].

Myeloid/lymphoid neoplasm with JAK2 rearrangement is included among myeloid/lymphoid neoplasms with eosinophilia and tyrosine kinase gene fusions (MLN‐TK) [4]. PCM1 is the most frequent fusion partner, and PCM1::JAK2‐positive neoplasms may show variable eosinophilia, myelofibrosis, and immature erythroid expansion [5, 6, 7].

NUTM1 rearrangements were initially regarded as defining alterations of aggressive NUT carcinoma but have increasingly been identified in diverse neoplasms [8, 9]. In haematological malignancies, they are best characterized in infant and paediatric B‐cell precursor acute lymphoblastic leukaemia with wild‐type KMT2A [10]. Here, we report an adult ES harbouring PCM1::JAK2 and a novel NOP10::NUTM1 fusion, complicated by secondary hemophagocytic lymphohistiocytosis.

## 2 Case Presentation

A 56‐year‐old woman was admitted to our hospital with a 2‐week history of fatigue accompanied by generalized myalgia, fever and night sweats. Laboratory evaluation showed hyperleukocytosis (64.47 × 10^9^/L), predominantly neutrophilic (neutrophils, 54.31 × 10^9^/L), with mild eosinophilia (1.26 × 10^9^/L), anaemia (haemoglobin, 88 g/L) and thrombocytopenia (55 × 10^9^/L). Peripheral blood smear showed left‐shifted granulopoiesis with 4% eosinophils. Bone marrow biopsy revealed myeloid and erythroid hyperplasia along with diffuse fibrosis and a mild increase in immature MPO‐ and CD71‐positive cells lacking CD117, E‐cadherin, and CD235a. Serum laboratory tests showed markedly elevated levels of lactate dehydrogenase (1806 U/L), ferritin (8551 μg/L), and soluble interleukin‐2 receptors (sIL‐2R; 7500 U/mL). Positron emission tomography‐computed tomography (PET‐CT) imaging revealed systemic lymphadenopathy with increased fluorodeoxyglucose (FDG) uptake and splenomegaly.

**FIGURE 1:**
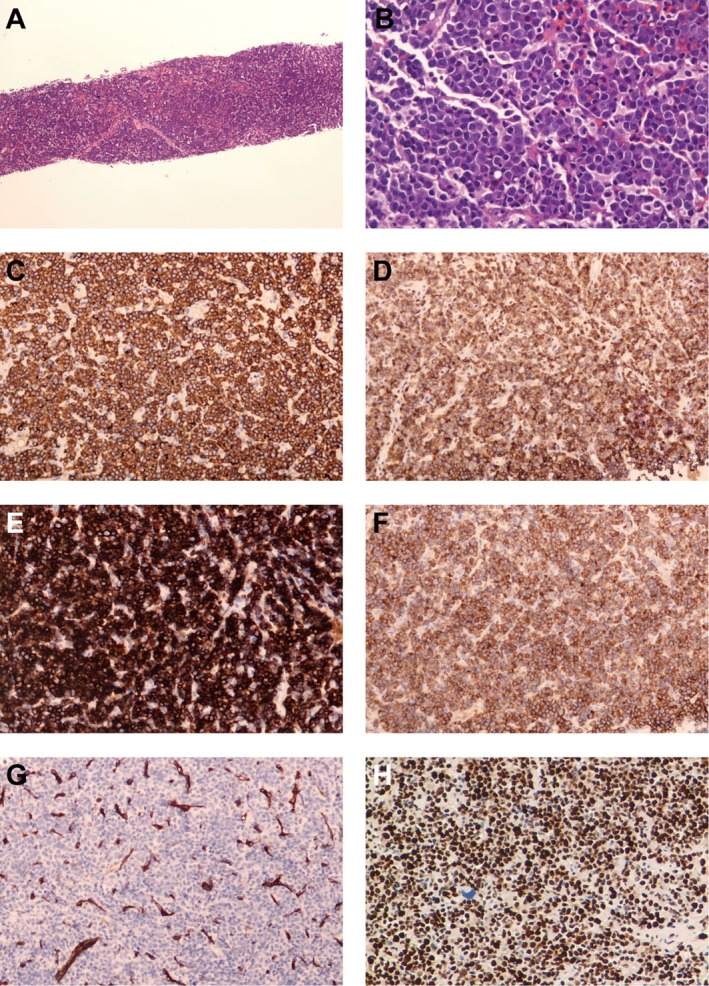
Histopathology of the biopsy specimen. (A and B) Haematoxylin and eosin (H&E)‐stained tissue sections. (A) The normal lymph node architecture was completely effaced and replaced by diffuse infiltration of immature cells. (B) Immature cells are medium‐ to large‐sized with fine chromatin, small and occasional nucleoli, and moderately abundant, mildly basophilic cytoplasm. Original magnification of objective lens ×10 (A) and ×40 (B). (C–H) Immunohistochemistry (IHC) results showing positive staining for CD71 (C), CD235a (D), CD43 (E), and E‐cadherin (F); negative staining for CD34 (G); and strong, diffuse positivity for p53 (H). Original magnification of objective lens ×20.

A left supraclavicular lymph‐node biopsy showed diffuse effacement by medium‐ to large‐sized immature cells with fine chromatin, occasional nucleoli, and moderately abundant cytoplasm (Figure 1A,B). The tumour cells expressed CD71, CD235a, CD43, and E‐cadherin (Figure 1C–F), with weak TdT and CD117 expression, whereas CD34 (Figure 1G), MPO, ERG, CD13, CD133 and the examined lymphoid/macrophage markers, including CD68, CD163, CD20, PAX‐5, CD3, CD5, CD7, CD56, CD123, and TCL1, were negative. Diffuse and strong p53 expression was observed in most tumour cells (Figure 1H). Flow cytometry identified an abnormal erythroblast population comprising 55.3% of nucleated cells, expressing CD71, CD235a/glycophorin A, and CD36, with partial CD105 and dim CD117/CD33 expression, supporting a diagnosis of ES (Figure S1).

**FIGURE 2:**
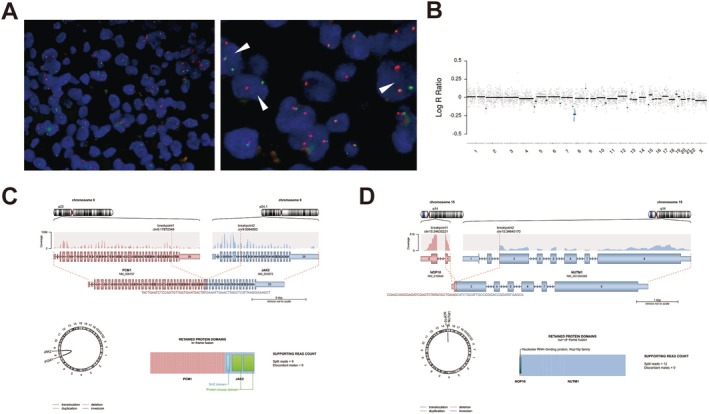
Cytogenetic and molecular analysis of the biopsy specimen. (A) JAK2 break‐apart fluorescence in situ hybridization analysis performed on formalin‐fixed, paraffin‐embedded tumour tissue. The normal signal pattern was 2F, whereas the rearranged pattern was 1F1R1G, consisting of one fused signal and separate red and green signals. Nuclei showing split red and green signals consistent with JAK2 rearrangement are indicated by white arrowheads. Among 200 evaluable interphase nuclei, 18% exhibited the 1F1R1G rearranged pattern, exceeding the predefined positivity cutoff of 10%. A dual‐colour JAK2 break‐apart probe, with the 5′ JAK2 region labelled in red and the 3′ JAK2 region labelled in green, was used according to the manufacturer's instructions (Wuhan Kanglu Biotechnology, China). F, fused red and green signal; G, green signal; R, red signal. (B) Genome‐wide copy‐number profile generated by shallow whole‐genome sequencing. A 17.0‐Mb copy‐number deletion involving chromosome 8p23.3–p22 was identified, with 17.2% of the sequencing fragments within this region supporting the copy‐number alteration (CNA). (C) Schematic representation of the *PCM1::JAK2* fusion transcript identified by whole transcriptome sequencing. An in‐frame fusion joined exon 36 of PCM1 to exon 9 of JAK2, with genomic breakpoints at chr8:17,872,349 and chr9:5,064,883, respectively. The fusion was supported by nine fusion‐supporting sequencing reads. (D) Schematic representation of the novel *NOP10::NUTM1* fusion transcript identified by whole transcriptome sequencing. An out‐of‐frame fusion involving exon 1 of *NOP10* and exon 3 of *NUTM1* at chr15:34635221 and chr15:34640. The fusion was supported by 12 fusion‐supporting sequencing reads.

JAK2 break‐apart fluorescence in situ hybridization (FISH) on the ES specimen demonstrated a 1F1R1G rearranged signal pattern in 18% of 200 evaluable interphase nuclei (Figure 2A), and whole‐transcriptome sequencing (WTS) of the same specimen identified an in‐frame PCM1::JAK2 fusion joining PCM1 exon 36 to JAK2 exon 9, with nine fusion‐supporting reads (Figure 2C). Shallow whole‐genome sequencing (sWGS) identified a copy‐number deletion involving chromosome 8p23.3–p22; 17.2% of the sequencing fragments within this region supported the copy‐number alteration (Figure 2B). WTS additionally detected a novel NOP10::NUTM1 fusion joining NOP10 exon 1 to NUTM1 exon 3, with 12 fusion‐supporting reads (Figure 2D), and a candidate NOTCH3 frameshift variant (p. T257Lfs*2; variant allele frequency 10.6%; sequencing depth, 189×), predicted to result in a truncated, likely loss‐of‐function protein. Bone marrow aspiration resulted in a dry tap because of fibrosis, precluding flow cytometric and molecular analyses of the marrow. Targeted next‐generation sequencing of the ES specimen using a myeloid neoplasm panel that included TP53 (Table S1) detected no reportable clonal pathogenic TP53 variant, and TP53/CEP17 FISH showed no TP53 deletion (Figure S2).

The patient met the diagnostic criteria for secondary hemophagocytic lymphohistiocytosis (HLH), with fever, bicytopenia, splenomegaly, hyperferritinemia, and elevated soluble interleukin‐2 receptor levels. She received etoposide and dexamethasone, followed by ruxolitinib, which achieved initial disease control. However, treatment was discontinued because of financial constraints, and she died of progressive disease 7 months after diagnosis.

## 3 Discussion

This case illustrates several diagnostic and biological issues in adult ES. The extramedullary lesion showed convincing erythroid differentiation, supported by CD71, CD235a, CD36, and E‐cadherin expression. However, leukocytosis with left‐shifted granulopoiesis, mild eosinophilia, marrow fibrosis, and immature MPO‐ and CD71‐positive marrow cells suggested an underlying myeloid process.

The relationship between the marrow abnormality and the extramedullary ES remains unresolved. Although the patient had no previously diagnosed myeloid neoplasm, the marrow fibrosis, mild eosinophilia, and the available marrow findings supported concurrent marrow involvement by a myeloid neoplasm. However, bone marrow aspiration resulted in a dry tap, precluding marrow flow cytometry and molecular analysis. Thus, the presence of PCM1::JAK2 or NOP10::NUTM1 in the marrow could not be assessed, and a shared clonal origin between the marrow and extramedullary disease could not be confirmed. The case may therefore represent erythroid blast transformation of an occult PCM1::JAK2‐positive MLN‐TK, but the temporal sequence of disease evolution remains uncertain.

Diffuse and strong p53 expression in more than 10% of tumour cells (Figure 1H) was consistent with the abnormal pattern reported in most adult ES cases (21/24, 88%) [3], but discordant with the absence of a reportable pathogenic TP53 alteration. Potential explanations include an unrecognised splice‐region, noncoding, intragenic, or structural TP53 alteration, mutation‐independent p53 stabilization, or, less likely, a very low‐level TP53‐mutated subclone below the reporting threshold.

The partial deletion of chromosome 8p is notable because it includes the PCM1 locus at 8p22. This copy‐number alteration may represent an unbalanced or complex structural event accompanying PCM1::JAK2 formation. However, sWGS cannot reconstruct derivative chromosome architecture; therefore, a direct mechanistic relationship between the deletion and fusion formation cannot be established.

PCM1::JAK2 rearrangement is a recognized driver in MLN‐TK, typically associated with eosinophilia, myelofibrosis, and immature erythroid precursors. Its identification in ES is uncommon but increasingly relevant. PCM1::JAK2 has now been reported in four adult ES cases, including this patient, suggesting a recurrent but uncommon association with erythroid transformation [11] (Table S2). Constitutive JAK2 activation provides a rationale for ruxolitinib, although responses may be transient and allogeneic haematopoietic stem‐cell transplantation remains the principal curative‐intent strategy when feasible [12]. In our patient, ruxolitinib was initiated after HLH‐directed therapy and achieved initial disease control, but treatment was discontinued because of financial constraints.

The novel NOP10::NUTM1 fusion expands the spectrum of NUTM1‐rearranged haematological neoplasms. NOP10 and NUTM1 are closely located at 15q14, making this intrachromosomal event potentially cryptic by conventional cytogenetics. A recent study indicated that *NUTM1* rearrangements in BCP‐ALL define a unique transcriptomic and epigenetic landscape, supporting recognition of a distinct leukaemia subtype [13]. By contrast, NUTM1 rearrangements are exceedingly rare in myeloid neoplasms. Reported partners include TIPIN, NAP1L4, AVEN, LARP1, ARHGAP15, and GABPB1, with most cases showing monocytic differentiation and poor outcomes [14] (Table S3). This finding highlights the value of RNA sequencing for detecting cryptic NUTM1 rearrangements in diagnostically challenging myeloid or erythroid neoplasms. However, the pathogenic significance of NOP10::NUTM1 in ES remains uncertain.

Malignancy‐associated HLH is a life‐threatening hyperinflammatory syndrome and rare in erythroid neoplasms. Only a few AEL‐associated secondary HLH cases have been reported, all with poor outcomes [15]. In this case, HLH may have contributed to the aggressive clinical course, although a direct mechanistic link cannot be established.

In summary, this case illustrates a molecularly complex adult ES with PCM1::JAK2, a novel NOP10::NUTM1 fusion, partial deletion of chromosome 8p, and secondary HLH. However, the absence of marrow fusion testing limits conclusions regarding clonal evolution. Integrated morphologic, immunophenotypic, cytogenetic, and sequencing analyses are essential for refining the diagnosis and clonal interpretation of adult ES.

## Author Contributions

**Tong Ge:** conceptualization, data curation, writing – review and editing, writing – original draft, visualization, formal analysis, investigation. **Dong Kuang:** validation, resources, data curation. **Xia Mao:** methodology, software, data curation. **Songya Liu:** methodology, data curation, visualization. **Jin Wang:** data curation, formal analysis. **Qilin Ao:** conceptualization, validation, supervision, resources. **Min Xiao:** funding acquisition, project administration, conceptualization, supervision, resources, writing – review and editing.

## Funding

This work was supported by the National Natural Science Foundation of China (No. 82270203 to Min Xiao) and the Science and Technology Major Project of Wuhan (No. 2025021102020385 to Min Xiao).

## Conflicts of Interest

The authors declare no conflicts of interest.

## Supporting information


**Figure S1:** Flow cytometric immunophenotype of the biopsy specimen, showing an abnormal erythroblast population supporting immature erythroid differentiation.
**Figure S2:** TP53/CEP17 fluorescence in situ hybridization analysis of the biopsy specimen, showing no TP53 deletion above the predefined cutoff.
**Table S1:** Targeted next‐generation sequencing panel used for molecular analysis of the biopsy specimen.
**Table S2:** Clinical and molecular features of reported patients with erythroblastic sarcoma harbouring PCM1::JAK2 fusion.
**Table S3:** Clinicopathologic and molecular features of reported myeloid neoplasms harbouring NUTM1 rearrangements.
**Methods S1**. Detailed methods for fluorescence in situ hybridization, shallow whole‐genome sequencing‐based copy‐number analysis, whole‐transcriptome sequencing, and targeted next‐generation sequencing.

## Data Availability

The data that support the findings of this study are available from the corresponding author upon reasonable request.
