# Supplementary material for: Adult Erythroblastic Sarcoma With PCM1::JAK2 Fusion and a Novel NOP10::NUTM1 Fusion Complicated by Secondary HLH
Source: J Cell Mol Med. 2026 Jul 15;30(14):e71286. doi: 10.1111/jcmm.71286 (PMC13373126; doi:10.1111/jcmm.71286)
Supplement: Supplementary file 1 — Figure S1: Flow cytometric immunophenotype of the biopsy specimen, showing an abnormal erythroblast population supporting immature erythroid differentiation. Figure S2: TP53/CEP17 fluorescence in situ hybridization analysis of the biopsy specimen, showing no TP53 deletion above the predefined cutoff. Table S1: Targeted next‐generation sequencing panel used for molecular analysis of the biopsy specimen. Table S2: Clinical and molecular features of reported patients with erythroblastic sarcoma harbouring PCM1::JAK2 fusion. Table S3: Clinicopathologic and molecular features of reported myeloid neoplasms harbouring NUTM1 rearrangements. Methods S1. Detailed methods for fluorescence in situ hybridization, shallow whole‐genome sequencing‐based copy‐number analysis, whole‐transcriptome sequencing, and targeted next‐generation sequencing. [file JCMM-30-e71286-s001.pdf]

Supplementary information (Methods, Figures and Tables)

Supplementary Methods

Fluorescence in situ hybridization

Fluorescence in situ hybridization (FISH) analysis was performed on formalin-fixed paraffin-embedded (FFPE) tissue sections using a *JAK2* dual-color break-apart probe (5′-JAK2, red; 3′-JAK2, green; Wuhan Kanglu Biotechnology, China) and a *TP53/CEP17* dual-color probe (TP53, red; CEP17, green; Wuhan Kanglu Biotechnology, China), according to the manufacturer's instructions. For each assay, 200 evaluable interphase nuclei were scored. Predefined cutoff values were 10% for JAK2 rearrangement and 20% for TP53 deletion.

Shallow whole-genome sequencing

LeukoPrint is a shallow whole-genome sequencing (sWGS)-based copy-number profiling assay used for genome-wide detection of copy-number alterations (CNAs), including CNAs of 1 Mb or larger(1). The procedures for DNA sample preparation and library construction have been previously described (2). The resulting sequencing data were used for genome-wide CNA analysis.

Whole transcriptome sequencing

FFPE tumor tissue was submitted to KingMed Diagnostics Laboratory for whole-transcriptome sequencing (WTS). RNA sample preparation and library construction were carried out as previously described(3). Candidate fusion transcripts and chimeric transcripts were detected using STAR-Fusion based on chimeric and split-read alignments generated by STAR.

Supplementary Figure 1.

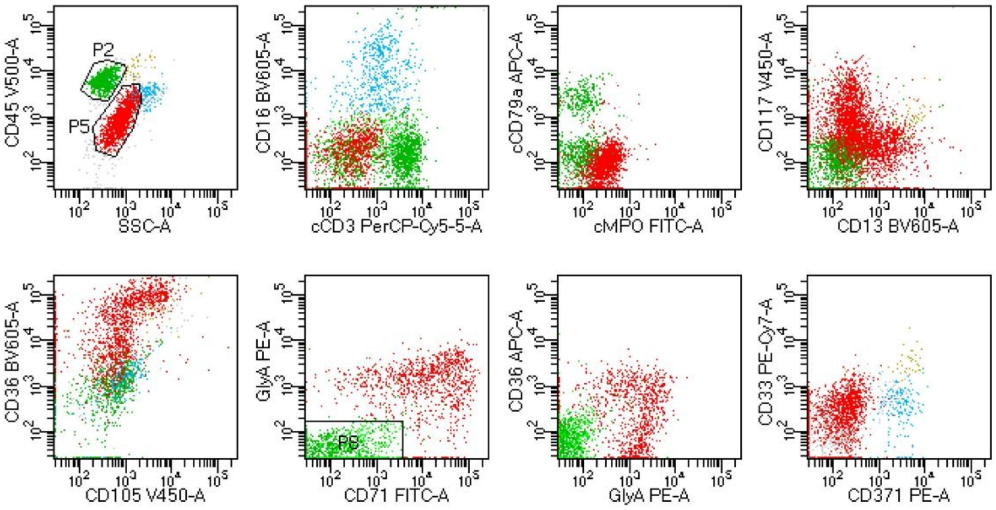

Supplementary Figure 1. Flow cytometric immunophenotype of the biopsy specimen.

The abnormal erythroblast population was consistently designated P5 and highlighted in red in all scattergrams. P5 accounted for 55.3% of the analyzed nucleated cells. This population showed an erythroid immunophenotype, with expression of CD71, glycophorin A/CD235a, and CD36, and partial expression of CD105. P5 showed low-to-dim CD45 expression and dim expression of CD117 and CD33, and was negative for CD371. Markers of T-cell, B-cell, granulocytic, and monocytic differentiation, including cytoplasmic CD3, CD16, CD79b, myeloperoxidase, and CD13, were negative. These findings support immature erythroid differentiation. GlyA, glycophorin A; MPO, myeloperoxidase.

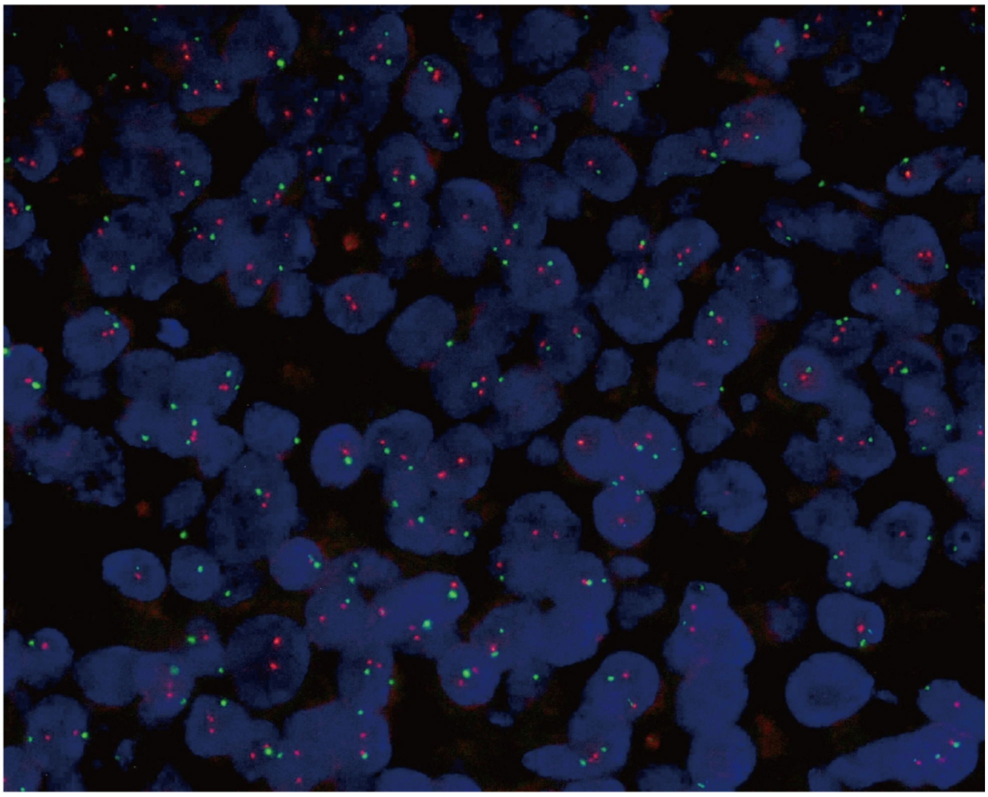

Supplementary Figure 2. Fluorescence in situ hybridization analysis for TP53 deletion.

Fluorescence in situ hybridization (FISH) was performed on formalin-fixed paraffin-embedded tissue sections using a dual-color TP53/CEP17 probe (TP53, red; CEP17, green). A total of 200 evaluable interphase nuclei were scored. A deletion-positive signal pattern was defined as one TP53 signal with two CEP17 signals, with a predefined cutoff of 20%. No TP53 deletion above the cutoff was detected in this specimen.

Supplementary Table 1. Targeted next-generation sequencing (NGS) panel.

ABLI	*ASXL1*	*ASXL2*	*BCOR*	*BCORLI*	*CALR*	*CBL*
CCND2	*CEBPA*	*CSF3R*	*DNMT3A*	*ETV6*	*EZH2*	*FLT3*
GATA2	*IDH1*	*IDH2*	*JAK2*	*JAK3*	*KIT*	*KMT2A*
KRAS	*MPL*	*NF1*	*NPM1*	*NRAS*	*PIGA*	*PPMID*
PTPN11	*RAD21*	*RUNX1*	*SETBP1*	*SF3B1*	*SH2B3*	*SRSF2*
STAG2	*STAT3*	*STAT5B*	*TET2*	*TP53*	*U2AF1*	*WTI*
ZRSR2						

Note: This panel covers 43 genes recurrently altered or clinically relevant in myeloid neoplasms. It interrogates the coding exons of these genes and selected clinically relevant non-coding regions. The panel is designed to detect single-nucleotide variants (SNVs) and small insertion/deletion mutations (indels).

Supplementary Table 2. Clinical and molecular features of reported patients with erythroblastic sarcoma harboring PCM1::JAK2 fusion.

**Case**	1	2	3	4
**Age, yrs**	32	72	32	56
**Sex**	M	M	M	F
**Initial diagnosis**	Myeloid neoplasm	MLN-Eo	Myeloid neoplasm	ES
**Biopsy site**	axillary LN	right posterior cervical LN	NA	supraclavicular LN
**Clinical presentations**	extensive LAD and splenomegaly	fatigue, abnormal CBC, LAD and splenomegaly	abnormal CBC, extensive LAD and splenomegaly	fever, fatigue, abnormal CBC, extensive LAD and splenomegaly
**Myelofibrosis**	Yes	Yes	NA	Yes
**Complication**	NA	NA	EBV+PTLD-DLBCL in LN	HLH
**Hemoglobin, g/L**	99	90	104	88
**Platelets, $\times 10^9$/L**	76	70	148	55
**Eosinophil, $\times 10^9$/L**	NA	2.12	NA	1.26
**Immunophenotype of the key immunostains**	E-cad (+), CD235a (+), CD117(+); CD45(-), CD34(-), MPO (-)	CD71(+), CD235a(+); CD34(-), MPO (-)	E-cad (+), CD235a (+), CD117(+, subset dim); CD45(-), CD34(-)	CD71(+), E-cad (+), CD235a (+), CD117(+, subset dim), p53(+); CD43(+); CD34(-), MPO (-)
**FISH**	BM: *PCM1::JAK2*<br>ES: *JAK2* rearrangement	BM: *PCM1::JAK2*	BM and ES: *PCM1::JAK2*	ES: *JAK2* rearrangement
**Mutations, VAF**	NA	BM: *RUNX1,*8%	BM: no clinically significant variants	ES: *NOTCH3* p.T257Lfs*2, 10.6%
**CNAs, AF**	NA	NA	NA	deletion of 8p23.2p22, 17.2%
**Other molecular features**	NA	NA	NA	*NOP10::NUTM1* fusion
**Method of Detection**	FISH	Karyotype, RT-PCR, FISH, NGS	Karyotype, FISH, NGS	FISH, NGS, sWGS, WTS
**SCT**	NA	no	yes	no
**Treatment**	NA	erythropoietin and prednisone	Vyxeos	etoposide, dexamethasone and ruxolitinib
**Follow-up (months)**	NA	12.3	8.5	7
**Outcomes**	NA	NA	Deceased	Deceased
**Report**	Luedke et al(4). 2020	Zhang et al(5). 2023	Fitzpatrick et al(6). 2025	Present case

BM: bone marrow; CBC: complete blood count; CNAs: copy number alterations; CR: complete remission; ES: erythroblastic sarcoma; F: female; FISH: fluorescence in situ hybridization; HLH: hemophagocytic lymphohistiocytosis; LAD: lymphadenopathy; LN: lymph node; M: male; MLN-Eo: myeloid/lymphoid neoplasms with eosinophilia; NA: not available; NGS: next generation sequencing; PTLD-DLBCL: Post-transplant lymphoproliferative disorder, diffuse large B-cell lymphoma subtype; RT-PCR: reverse transcription polymerase chain reaction; SCT: stem cell transplantation; sWGS: shallow whole-genome sequencing; VAF: variant allele frequency; WTS: whole transcriptome sequencing.

Supplementary Table 3. Clinicopathologic and molecular features of reported myeloid neoplasms harboring NUTM1 rearrangements.

Age	Sex	Diagnosis	Status	Partner gene	Fusion type	5' breakpoint	Exon	3' breakpoint	Exon	Intra-chromosomal fusion	Karyotype	Mutations	Other fusions	Outcomes	Report
82	M	AML-M4	NA	TIPIN	5'UTR	chr15: 66648977	NA	chr15: 34640168	NA	yes	48-49,XY,+8,+del(8)(q22),del(13)(q11),+del(13)(q11),der(16)t(16;21)(p13;q?),der(17)t(13;17)(q22;p11)[cp13]/96,XXYY,+8,+8,+8,+8,+9,-10,r(10)(?),del(13)(q11)x2,+del(13)(q11),der(16)t(16;21)(p13;q?),-17,del(17)(p11)x2[2]	NA	RUNX1::THOC, MAP2K1::TIPIN	NA	Stengel et al(7), 2020
27	M	MLN-Eo	ND	NAP1L4	NA	NA	Exon9	NA	Exon5	no	t(11;15)(p15;q12)[18]/46,XY [2]	NA	FIP1L1::PDGFRα	complete remission	Cheng et al(8), 2020
43	F	AML-M4	R/R	ATEN	in-frame	NA	Exon2	NA	Exon2	yes	46,XX,der(20)t(2;20)(p13;p13)	RUNX1 (L98Sfs*24), IDH1 (R132C)	NA	relapsed post-transplant	Yuan et al(9), 2021
65	M	AML	R/R	LARP1	in-frame	chr5: 154135753	Exon1	chr15: 34640170	Exon2	no	46,XY,t(3;11;6)(p21;p15;q23),t(5;15)(q33;q11.2)[18]/46, XX[2]	ASXL2 (K873fs*6), BCOR (S1297*), DNMT3A (D845fs*8), IDH2 (R172K), NSD1 (V1016fs*27)	NA		
67	M	AML	R/R	ARHGAP15	in-frame	chr2: 143986237	Exon5	chr15: 34638143	Exon1	no	t(2;15) (q23;q15)[9]/46,XY[11]	ASXL1 (G646fs*12), RUNX1 (R169fs*44), TET2 (Q1942*)	NA	relapsed post-transplant and deceased	Tizro et al(10), 2025
72	M	AML	R/R	GABPB1	in-frame	chr15: 50647182	Exon1	chr15: 34640170	Exon2	yes	46, XY, i(7)(p10),t(4;21)(q12;q22), del(13)(q14q22)[16]/46, XX[4]	AMER1 (R1049*), BCOR (K395fs*47), DNMT3A (R882H), GATA2 (R362Q), IDH1 (R132C), PHF6 (Q37*), NF1 loss	NA		
56	F	ES	ND	NOP10	out-of-frame	chr15: 34635221	Exon1	chr15: 34640170	Exon3	yes	BM	NOTCH3 (T257Lfs*2)	PCM1::JAK2	deceased	Present case

AML: acute myeloid leukemia; BM: bone marrow; ES: erythroblastic sarcoma; F: female; M: male; MLN-Eo: myeloid/lymphoid neoplasms with eosinophilia; NA: not available; ND: newly diagnosed; R/R: relapsed/refractory.

Reference

1. Fang B, Zhu Z, Chang Y, He X, Li S, Li M, et al. Genome-wide copy number profiling enhances risk stratification in multiple myeloma by shallow whole-genome sequencing. Blood Adv. 2025.
2. Lyu X, Li T, Zhu D, Cheng Y, Chen Y, He X, et al. Whole-genome sequencing as an alternative to analyze copy number abnormalities in acute myeloid leukemia and myelodysplastic syndrome. Leuk Lymphoma. 2022;63(10):2301-10.
3. Bao M, Zhang XS, Li ZR, Yu L, Gale RP, Zhao SS, et al. Efficacy, safety and predictive biomarker of third-generation tyrosine kinase inhibitors with azacitidine in myeloid blast phase of chronic myeloid leukemia. Cancer. 2025;131(22):e70166.
4. Luedke C, Rein L. Transformation to erythroblastic sarcoma from myeloid neoplasm with PCM1-JAK2. Blood. 2020;136(9):1113.
5. Zhang L, Zhu X, Qu W, Lu Y, Feng Z, Zhao L. Myeloid/lymphoid neoplasms associated with eosinophilia and rearrangements of PCM1::JAK2 with erythroblastic sarcoma: a case report and literature review. Haematologica. 2023;108(12):3506-10.
6. Fitzpatrick MJ, Yuan J, Capa I, Bledsoe JR, Kibler CE, Tucker C, et al. Erythroblastic Sarcoma in Adults and Children: Different Pathways to the Same Destination. Mod Pathol. 2025;38(8):100716.
7. Stengel A, Shahswar R, Haferlach T, Walter W, Hutter S, Meggendorfer M, et al. Whole transcriptome sequencing detects a large number of novel fusion transcripts in patients with AML and MDS. Blood Adv. 2020;4(21):5393-401.
8. Cheng Z, Luo Y, Zhang Y, Wang Y, Chen Y, Xu Y, et al. A novel NAP1L4/NUTM1 fusion arising from translocation t(11;15)(p15;q12) in a myeloid neoplasm with eosinophilia and rearrangement of PDGFRA highlights an unusual clinical feature and therapeutic reaction. Ann Hematol. 2020;99(7):1561-4.
9. Yuan L, Chen X, Cao X, Wang F, Zhang Y, Ma X, et al. Identification of a novel AVEN-NUTM1 fusion gene in acute myeloid leukemia. Int J Lab Hematol. 2021;43(4):O207-O10.
10. Tizro P, Chang L, Salhotra A, Arias-Stella J, Telatar M, Tomasian V, et al. Novel NUTM1 Fusions in Relapsed Acute Myeloid Leukemia: Expanding the Genetic and Clinical Landscape. Int J Mol Sci. 2025;26(23).
